# A portable pen-sized instrumentation to measure stiffness of soft tissues in vivo

**DOI:** 10.1038/s41598-020-79735-8

**Published:** 2021-01-11

**Authors:** Zhengwei Li, Alireza Tofangchi, Robert A. Stavins, Bashar Emon, Ronald D. McKinney, Paul J. Grippo, M. Taher A. Saif

**Affiliations:** 1 Department of Mechanical Science and Engineering, University of Illinois at Urbana-Champaign, Urbana, IL 61801 USA; 2 Department of Medicine, Division of Gastroenterology and Hepatology, University of Illinois at Chicago, Chicago, IL 60612 USA; 3 Department of Bioengineering, University of Illinois at Urbana-Champaign, Urbana, IL 61801 USA

**Keywords:** Engineering, Biomedical engineering, Mechanical engineering

## Abstract

Quantitative assessment of soft tissue elasticity is crucial to a broad range of applications, such as biomechanical modeling, physiological monitoring, and tissue diseases diagnosing. However, the modulus measurement of soft tissues, particularly in vivo, has proved challenging since the instrument has to reach the site of soft tissue and be able to measure in a very short time. Here, we present a simple method to measure the elastic modulus of soft tissues on site by exploiting buckling of a long slender bar to quantify the applied force and a spherical indentation to extract the elastic modulus. The method is realized by developing a portable pen-sized instrument (EPen: Elastic modulus pen). The measurement accuracies are verified by independent modulus measures using commercial nanoindenter. Quantitative measurements of the elastic modulus of mouse pancreas, healthy and cancerous, surgically exposed but attached to the body further confirm the potential clinical utility of the EPen.

## Introduction

Considerable progress has been made over the past two decades in measuring elastic modulus of hard materials at macro^1,2^, micro^3,4^ and nano^5,6^ scales. However, there is a long-term unmet need for measuring the local elastic modulus of soft materials and tissues on site, including cell culture substrates for mechanobiological studies^7^ and drug screening^8^, engineered scaffolds for tissue engineering^9^, bone regeneration^10^ and biohybrid machines^11,12^, and in situ healthy and pathological tissues for disease diagnosis and prognosis^13,14^. In contrast to nonliving hard materials, most soft biomaterials and tissues, engineered or in situ, do not lend themselves to fabrication of test specimens with standard geometry used by current material testing instruments. In addition, there are many challenges associated with the elastic modulus measurements. For example, the methods to measure their modulus need to satisfy the following conditions in regards to the instrument: (1) has to reach the site of the materials or tissues (not the other way around), therefore, the instrument has to be portable and preferably hand-held, and (2) must be able to measure the modulus in a very short time to preserve the change of mechanical properties, particularly for the soft tissues after their surgical resection from the body or after opening the body during a surgical procedure, and (3) should be versatile for a wide range of stiffness measurements for different types of soft tissues or different stages of pathological tissues (e.g. malignant tumors)^15^. Currently, there are no method available to meet these criteria.

Indentation^16,17^ has been widely used to measure the mechanical properties of biological tissue systems. Much of the reported literature work focused on the skin and subcutaneous tissues, such as human forehead skin^18^, residual limbs^19^, fibrosis neck tissues^20^ and plantar foot tissues^21^. Several generations of indentation instruments have been developed which usually employ a load cell to measure the loading force and a displacement transducer to record the tissue deformation^22–25^. However, the structures of their mechanical indentation apparatuses are either complicated or large which are not convenient for in vivo tests, such as that of soft pancreatic organ. Some portable, hand-held indentation instruments^26–28^ have also been reported. For example, an ultrasound indentation system^26^ has been developed for measuring biomechanical properties of plantar-foot tissues and breast tissue in vivo. A portable pen sized indentation probe^27^ has been reported by tracking the ultrasound echo that is reflected from the substrate bone to measure the tissue deformation. However, these ultrasound indentation systems are very expensive, sensitive to the noise, and not suitable for the measurements of thick tissues due to the attenuation of ultrasound^28^. Furthermore, almost all current indentation systems have a fixed stiffness of the indenter which greatly hinder their usage for diverse modulus measurements.

Here, we propose a simple and novel method for measuring elastic modulus of soft materials or tissues on site. Such method is based on two well-established theories of mechanics: (1) buckling of a long slender bar, where the critical threshold force is determined by its geometry and elastic properties, and (2) Hertz contact theory between a hard sphere and soft materials, which offers the relationship between the applied force and deformation (i.e., indentation) of the soft materials or tissues. Our method exploits buckling to quantify the applied force on the hard sphere, and spherical indentation on the soft materials to extract the elastic modulus. The method is realized by developing a hand-held portable instrument (EPen: Elastic modulus pen) with a wide measurement range, and validated by measuring elastic modulus of Polyacrylamide (PA) gel with known modulus values (0.5, 2.5 and 5 kPa). The measured elastic modulus by the EPen matches well with those by both commercial nanoindenter and standard fabrication protocol. The potential clinical application of our EPen is further demonstrated by measuring the modulus in situ in living tissues: healthy and cancerous pancreatic tissues of healthy and KPC mice respectively.

## Results

### Theory

**Figure 1:**
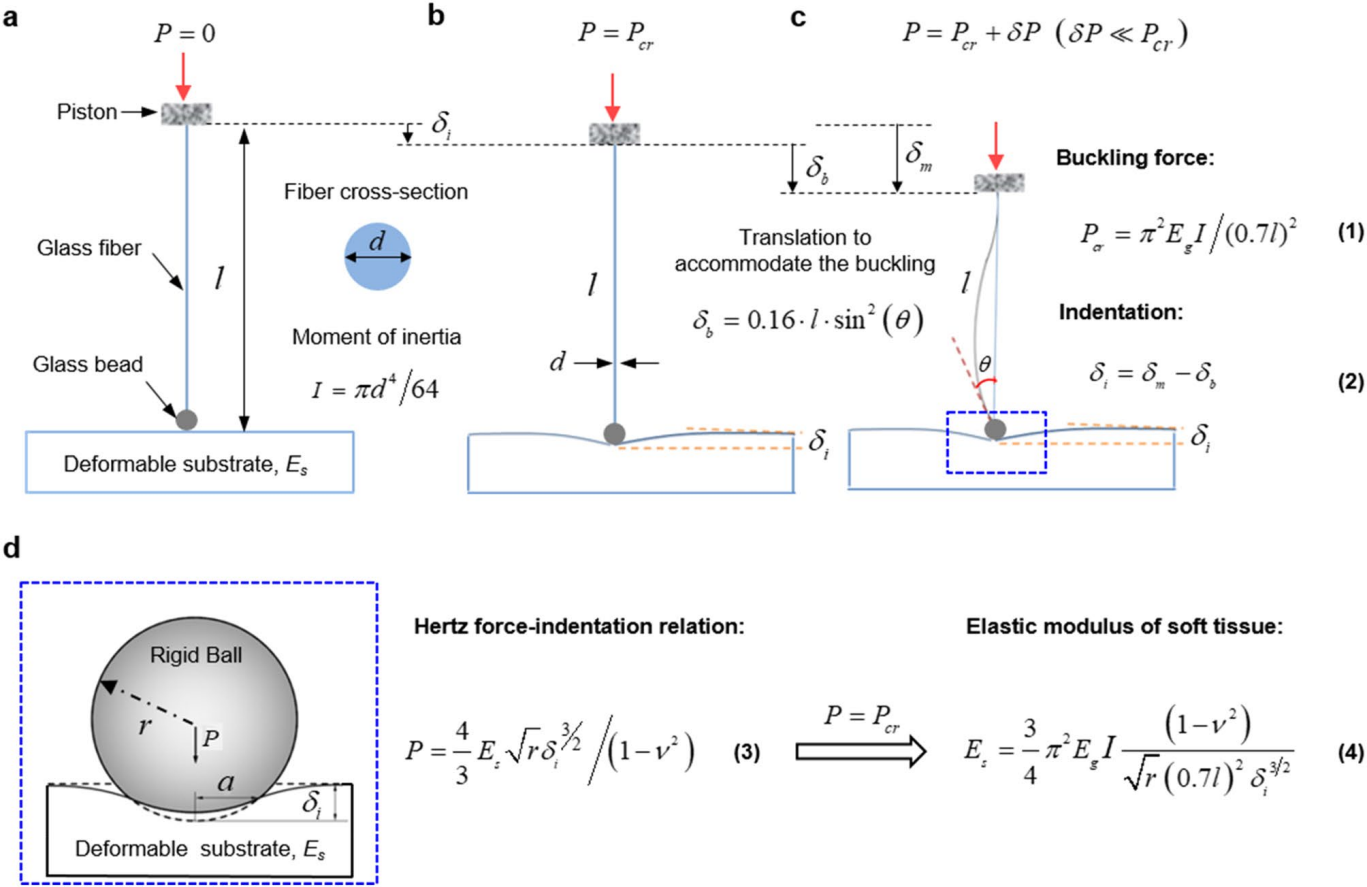
Theory of buckling and Hertzian indentation. (**a**) Schematic illustration of a slender glass fiber with one end held by a movable piston and the other end fixed to a rigid glass sphere, which serves as a probe to measure the stiffness of soft materials or tissues. (**b**) Compression controlled by a piston induces the critical buckling state of the glass fiber, where the displacement of moving piston equals to the indentation amount $\delta_{i}$ of the substrate. (**c**) Further compression on the glass fiber results in the fiber buckling with a rotation angle $\theta$ at one end. (**d**) Schematic of the spherical indentation on the sample, where the indentation is used to extract the elastic modulus.

The method exploits two well-established theories of mechanics to measure the elasticity of the soft materials or tissues (Fig. 1). (1) *Buckling of long slender bar:* a bar subjected to an axial compressive force $P$ at both ends buckles when the force exceeds a critical threshold value $P_{cr}$. When $P > P_{cr}$, the bar continues to buckle with very small increase of force $\delta P$($\delta P$ <  < $P_{cr}$) due to the ignorable axial stiffness^29,30^. Therefore, $P_{cr}$ can be considered as the force imposed by the slender bar at any post buckled state. This introduces a small error in the measurement of elastic modulus, yet can be quantified (See Supplementary Note S1). (2) *Hertz contact theory*: a hard sphere in contact with an elastic material subjected to a compressive force deforms the material or indents it. If the deformation is small, the soft material recovers to its original configuration upon removal of the force, and the amount of indentation depends on the contact load, geometry of sphere and elasticity of the indented material.

Consider a long slender bar of length $l$ and dimeter $d$ with one end fixed to a solid glass bead of radius $r$ and the other end held by a movable piston, as shown in Fig. 1a. Firstly, the sphere is brought in contact with the surface of a soft sample. Then, the piston is used to push the bar towards the sample until the sphere indents the sample by $\delta_{i}$ when the bar begins to buckle (Fig. 1b). The critical buckling force $P_{cr}$ is determined by the geometry and elasticity of the bar and given by,

1 $$P_{cr} = {{\pi^{2} E_{g} I} \mathord{\left/ {\vphantom {{\pi^{2} E_{g} I} {(0.7l)^{2} }}} \right. \kern-\nulldelimiterspace} {(0.7l)^{2} }}$$

where the moment of inertia, $I = {{\pi d^{4} } \mathord{\left/ {\vphantom {{\pi d^{4} } {64}}} \right. \kern-\nulldelimiterspace} {64}}$ and $E_{g}$ is the elastic modulus of glass fiber. When $P > P_{cr}$, the bar continues to buckle with a buckling angle $\theta$ with a small increase of force $\delta P$ (Fig. 1c). Hence, $P_{cr}$ could be considered simply as the force supported by the bar at any post-buckling state. The total translation of the piston from the initial contact to buckling is $\delta_{m} = \delta_{i} + \delta_{b}$, where $\delta_{b}$ is the part of the translation needed to accommodate the buckling (Fig. 1b) which can be determined from the deformed shape of the bar after buckling. Once the length $l$ is given, $\delta_{b}$ can be obtained analytically in the function of buckling angle $\theta$, i.e., $\delta_{b} = 0.16 \cdot l \cdot \sin^{2} \left( \theta \right)$ (See Supplementary Note S2). Therefore, indentation $\delta_{i}$ on the soft materials or tissues is obtained from $\delta_{m}$ and $\delta_{b}$ by,

2 $$\delta_{i} = \delta_{m} - \delta_{b}$$

Although the bar shortens elastically by a small amount due to the axial force, the axial shortening is negligible compared to the displacement $\delta_{b}$ due to the buckling (see Supplementary Note S3).

Hertz contact theory is then used to extract the elastic modulus of soft material based on the indentation on the soft materials or tissues. A hard sphere of radius $r$ contacting an elastic substrate with a contact force $P$, deforms the material elastically by $\delta_{i}$, as long as the strains induced are within elastic limit of the indented material (Fig. 1d). The force-indentation relation is given by

3 $$P = {{\frac{4}{3}E_{s} \sqrt r \delta_{i}^{{{\raise0.7ex\hbox{$3$} \!\mathord{\left/ {\vphantom {3 2}}\right.\kern-\nulldelimiterspace} \!\lower0.7ex\hbox{$2$}}}} } \mathord{\left/ {\vphantom {{\frac{4}{3}E_{s} \sqrt r \delta_{i}^{{{\raise0.7ex\hbox{$3$} \!\mathord{\left/ {\vphantom {3 2}}\right.\kern-\nulldelimiterspace} \!\lower0.7ex\hbox{$2$}}}} } {\left( {1 - \nu^{2} } \right)}}} \right. \kern-\nulldelimiterspace} {\left( {1 - \nu^{2} } \right)}}$$

where $E_{s}$ and $\nu$ are the elastic modulus and Poisson’s ratio of the soft material. It is known that most soft materials and tissues remain linearly elastic up to a strain of 10%^31,32^, and have Poisson’s ratio between 0.4 and 0.5^33,34^. We use $\nu = 0.5$ for all materials to be tested similar to other standard nanoindenter testing machines (e.g. Piuma nanoindenter). It should be noted that this assumption introduces a small error in the measurement of elastic modulus $E$. Alternatively, one can consider ${E \mathord{\left/ {\vphantom {E {(1 - }}} \right. \kern-\nulldelimiterspace} {(1 - }}\nu^{2} )$ as an effective elastic modulus of the material without introducing any value for $\nu$. With the known values of applied force and indentation, we can evaluate the elastic modulus of soft tissues by

4 $$E_{s} = {{\frac{3}{4}\pi^{2} E_{g} I(1 - \nu^{2} )} \mathord{\left/ {\vphantom {{\frac{3}{4}\pi^{2} E_{g} I(1 - \nu^{2} )} {\sqrt r }}} \right. \kern-\nulldelimiterspace} {\sqrt r }}(0.7l)^{2} \delta_{i}^{{{\raise0.7ex\hbox{$3$} \!\mathord{\left/ {\vphantom {3 2}}\right.\kern-\nulldelimiterspace} \!\lower0.7ex\hbox{$2$}}}}$$

It should be noted that our method exploits buckling as a threshold force readout and avoids using any force sensor.

### Implementation

**Figure 2:**
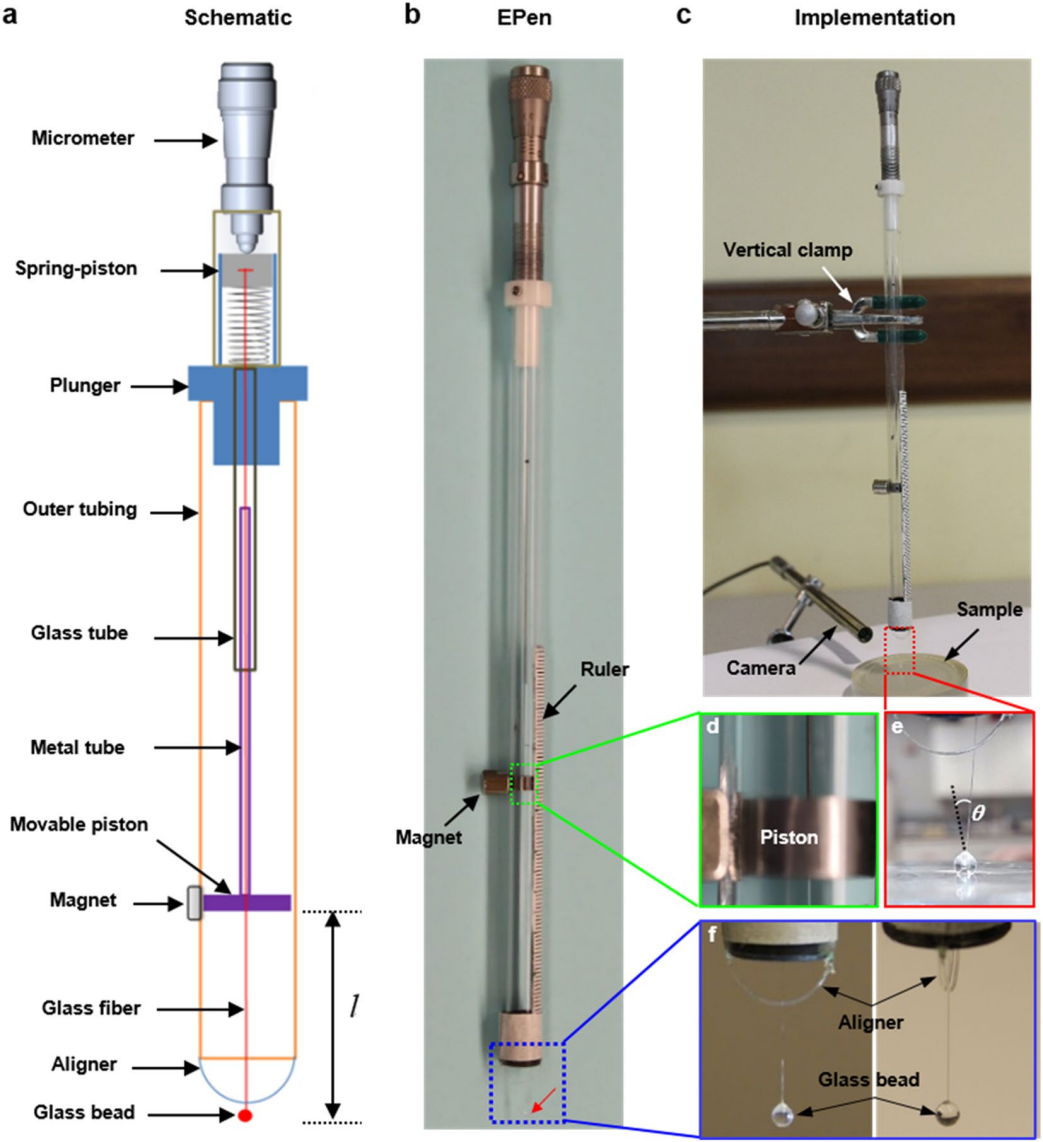
Fabrication and implementation. (**a**) Schematic of the elastic modulus pen (EPen) with each component indicated (drawn by using AutoCAD 2017, https://www.autodesk.com). (**b**) Optical image of the fabricated EPen. (**c**) Implementation of EPen in the experiment to measure the stiffness of soft samples, where the EPen is held vertically by using a clamp. And a camera is used to monitor the contact and fine motion of the glass bead. (**d**) Magnified optical image of movable position controlled by an external magnet. (**e**) The buckling of glass fiber with a rotation angle $\theta$ in the experiment. (**f**) A pair of arc shaped wires are attached to the EPen body to guide the direction of buckling of the glass fiber.

The method is realized by developing a portable pen-sized instrument that is EPen: Elastic modulus pen (Fig. 2a,b). It consists of a long slender glass fiber with 80 μm diameter encased in a glass/acrylic hollow cylinder (i.e. outer tubing) which serves as the body of the EPen (Fig. 2a). One end of the glass fiber is fixed to a glass bead/sphere with 2 mm diameter, and the other end is connected to a spring-loaded piston, the position of which can be precisely controlled by a Vernier micrometer. Two concentric tubes (i.e. glass tube and metal tube) are used to precisely align the glass fiber along the axial direction, as well as to selectively create “effective length” for buckling and thus an adjustable stiffness. The glass tube is fixed at its upper end to the plunger section, while the metal tube is attached at its lower end to a stainless steel piston enclosed in the outer acrylic cylinder tubing. The motion of steel piston (Fig. 2d) is controlled by a small magnet outside the enclosure adjacent to it, which can move the metal tube inside the concentric glass tube. This unique feature enables to create variable effective glass fiber length for the buckling and thus adjustable stiffness, with one end clamped near the piston at desired position and the other end probing the substrate. Longer the glass fiber length, lower is the critical buckling load. We made the length as an adjustable parameter so that the same EPen can be used to measure elastic modulus of samples with diverse stiffness. A pair of arc-shaped spacer/aligner (Fig. 2f) are further designed and attached to the bottom end of EPen body to guide and retain the plane of buckling for the sake of consistency and accurate optical imaging of the glass fiber.

To measure the elastic modulus, the EPen is held vertically by a clamp (Fig. 2c). The sample is brought in proximity of the glass sphere by adjusting the clamp height. Once approached close enough, the Vernier micrometer of the EPen is then used to bring the glass sphere in contact with the sample. And a camera is used to visualize the fine motion of the sphere and monitor the contact. The micrometer is then used to move the glass fiber further by $\delta_{m}$ (Fig. 1c) until the bar buckles with a buckling angle $\theta$ that can be obtained directly based on the image of buckled glass fiber (Fig. 2e). The total effective length of glass fiber $l$ can readily be determined based on a scale ruler in mm range (Fig. 2b) attached to the EPen body. The total translation $\delta_{{\text{m}}}$ of the glass fiber is directly determined by taking two measurements of the Vernier micrometer by the buckling bar before and after application of force on the sample. The buckling rotation angle $\theta$ is obtained by two images of the glass fiber taken before and after the buckling, which gives $\delta_{b}$. Thus, the indentation $\delta_{i}$ is obtained by $\delta_{{\text{m}}}$-$\delta_{b}$, which provides the elastic modulous of soft materials or tissues. Therefore, the entire testing process involves taking two measurements and two images, which takes less than 1 min.

### Calibration

**Figure 3:**
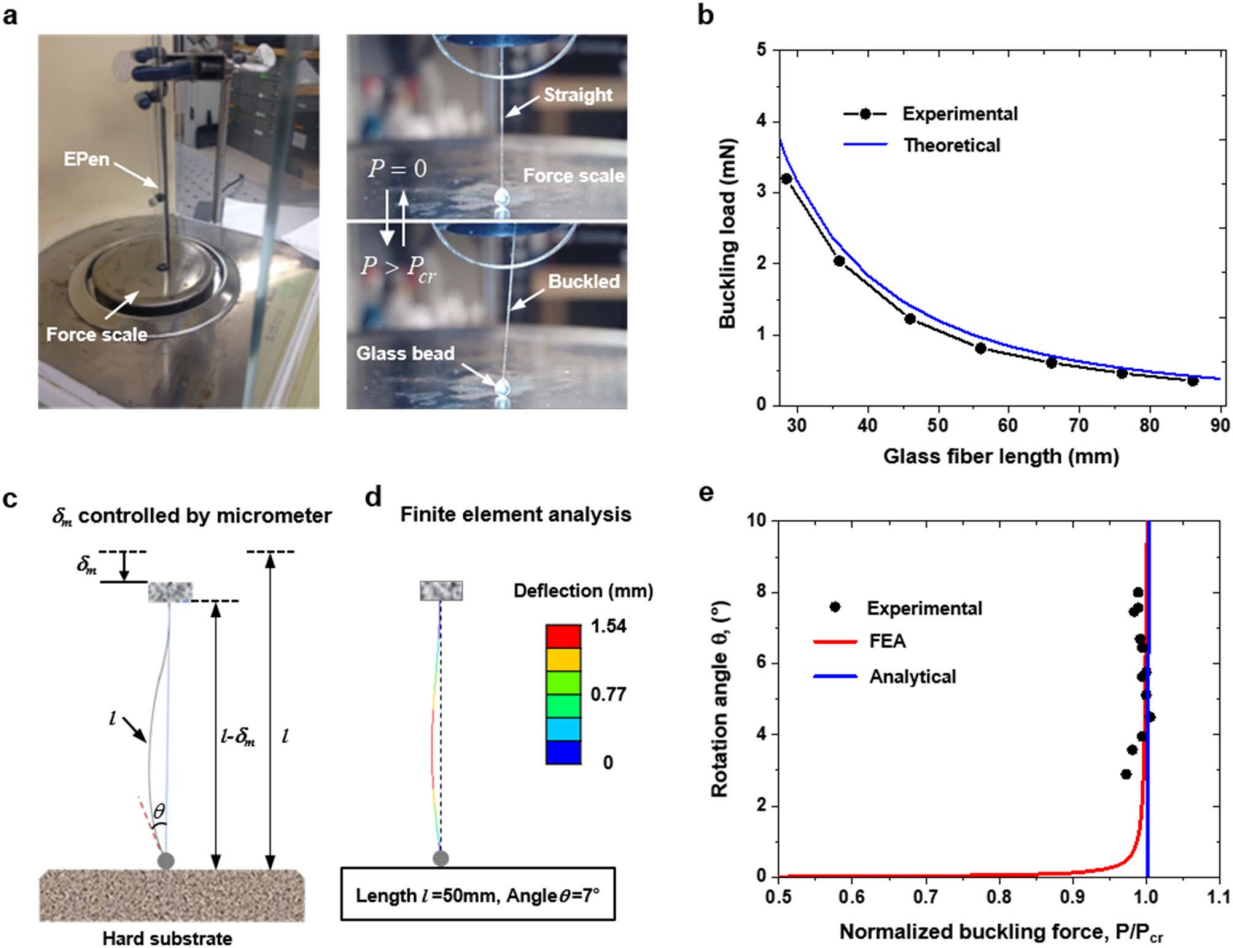
Calibration and error analysis. (**a**) The measurement of critical buckling force by buckling the glass fiber against a force scale. (**b**) The relation between the critical buckling force and glass fiber length is shown. Experimental values are slightly lower due to unavoidable imperfections in the fiber and misalignment between the fiber and the sphere. (**c**) Schematic of the buckled glass fiber on a hard substrate by a displacement control via a micrometer. (**d**) The deflection contours of a slender glass fiber with the initial length of 50 mm and buckling angle 7°. (**e**) Measurements of rotation angle as a function of applied force obtained from the experiment (black dots) are quantitatively consistent with analytical calculations of the mechanics (analytical; blue line) and finite element analysis (FEA, red line).

The critical buckling load $P_{cr}$ of glass fiber in EPen is given by Eq. (1). However, the load is for an ideal bar without any imperfection, i.e., there is no initial curvature in the fiber (perfectly straight) and the contact force between the glass sphere and glass fiber is aligned with its longitudinal axis. In reality, these conditions might be violated, and the glass fiber may show transverse deformation before the force is applied. Hence, the glass fiber with a sphere need to be calibrated experimentally for its “real” force–deformation relation. We calibrate the glass fiber with a prescribed length by a force sensor, namely a micro balance (Fig. 3a). We use the EPen to apply force on the balance until the bar buckles along the transverse direction. The critical buckling force $P_{cr}$ can be directly read from the micro balance. By calibrating the buckling load for different glass fiber length $l$, we get a relation between $l$ and $P_{cr}$(Fig. 3b). Corresponding theoretical relation is also shown in Fig. 3b. As expected, the theoretical prediction is slightly higher (8.2–15.5% within the fiber lengths considered) than the experimental values, since the theory does not account for (a) any imperfection in the buckling bar and (b) any misalignment between the bar and the glass sphere. In reality, these conditions are violated. Therefore, we calibrated the buckling load for different glass fiber lengths.

When $P > P_{cr}$, the bar continues to buckle with very small increase of force $\delta P$($\delta P \ll P_{cr}$). Simply considering $P_{cr}$ as the applied force will introduce a small error in the measurement of elastic modulus. Therefore, we further investigate the effect of buckling angle $\theta$ on the applied force both experimentally and theoretically. The force scale is again used to measure the applied force with the change of buckling angles. Figure 3c shows the schematic illustration of the measuring process. It should be noted that the indentation $\delta_{i}$ is zero for the solid surface, so $\delta_{{\text{m}}}$ equals to $\delta_{b}$. The accuracy of our experimental measurements, shown by the black dots in Fig. 3e, is further validated by both finite element analysis (FEA) (Fig. 3d and Supplementary Fig. S4a) and analytical buckling analysis (see Supplementary Note S1). The results show that the estimations of $P = P_{cr}$ results in up to 5% error as the buckling angle is below 5°, yet the error decreases as small as 2% when the buckling angle exceeds 5° (Fig. 3e). Therefore, we allow the glass fiber to deflect up to 5° before the read out for sake of accuracy and ease of optical recording.

### Validation

**Figure 4:**
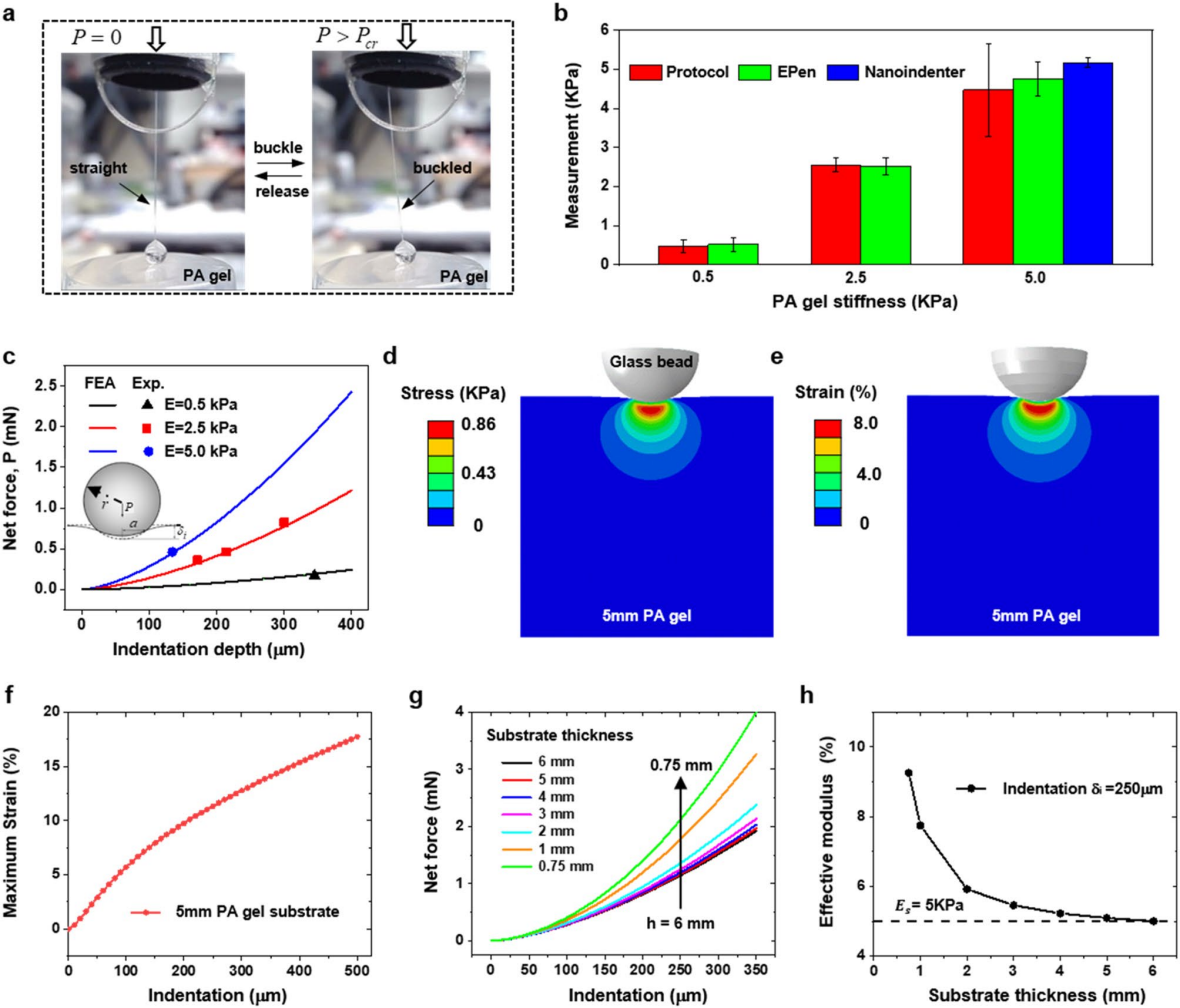
Testing and validation. (**a**) Optical images of glass bead of the EPen to probe the modulus of PA gel by harnessing the buckling of glass fiber. (**b**) Comparison between the stiffness measurements of 0.5, 2.5 and 5 kPa PA gel obtained by fabrication protocol, EPen and nanoindenter respectively. (**c**) Measurements of net force as a function of indentation depth obtained from the experiment (dots) which agrees very well with FEA results (lines) for 0.5, 2.5 and 5 kPa PA gel respectively. (**d**,**e**) The stress and strain contours of 5 mm PA gel with elastic modulus of 5 kPa under the spherical indentation of 134 μm. (**f**) Maximum strain in the PA gel substrate depending on the spherical indentation. (**g**) Net indentation force versus the spherical indentation amount with various substrate thickness. (**h**) Simulated effective modulus depending on the substrate thickness.

We use the EPen to measure the elastic modulus of PA gel with different known moduli to validate the accuracy (Fig. 4a). The gel samples are prepared by curing liquid PA gel in 3D printed circular wells of 25 mm in diameter and 5 mm in depth, following the standard fabrication protocol^35^. We prepare the PA gels with nominal elastic moduli of 0.5, 2.5, and 5 kPa, and Poisson’s ratio is assumed to be 0.5. Nanoindenter (Optics Piuma, 2015) with spherical tip of 25 µm radius is firstly used to measure the prepared 5 kPa gel samples. The measured modulus by nanoindenter is 5.16 kPa verifying the accuracy of the fabrication protocol. EPen is then used to measure the elastic moduli of each gel sample at 5 to 8 different positions. The measured modulus by EPen for 0.5, 2.5, and 5 kPa PA gel samples are all very close (within 10%) to the values obtained by standard fabrication protocols (Fig. 4b).

FEA is further used to study the indentation mechanics and simulate the spherical indentation on PA gel substrates with different elastic modulus. Figure 4c gives the net indentation force,$P$ versus the indentation depth,$\delta_{i}$, by both FEA and the EPen. FEA results agree very well with the experiment, verifying the accuracy of the EPen measurements. We also find that the boundaries of stress field induced by the indentation stay far away from the bottom surface of PA gel (Fig. 4d) and maximum strain level remains under 10% (Fig. 4e) when the spherical indentation is 134 µm, similar to the EPen measurements for 5 kPa PA gel in the experiment. However, as the indentation increases to 500 µm, the maximum strain in the substrate increases to 17.7% due to the boundary effect (Fig. 4f). In the EPen measures, the indentation depths $\delta_{i}$ are restricted to 250 µm to ensure the elastic strains below 15% to avoid the effect of bottom boundary (See Supplementary Note and Supplementary Fig. S5). Furthermore, we find that thinner substrate requires higher applied net force (Fig. 4g), causing a higher effective modulus deviating from the actual values (Fig. 4h). FEA results also show that the friction or adhesion between the glass sphere and PA gel substrate does not affect the net indentation force (Supplementary Fig. S6). Here, the effect of adhesion between the sphere and the substrate on the indentation load was simulated by introducing no-slip condition. The results show that the adhesion does not noticeably affect the indentation force, especially when the indentation depth is small (SI Fig. 6). Finally, we performed a 3D finite element analysis to investigate the effect of adhesion on the indentation force as the sphere rotates after buckling. Here, we applied an adhesive contact condition between the sphere and soft substrate. Thus, there is no separation or sliding between them. We used a 2 mm diameter glass sphere to indent 5 kPa substrate by 150 µm. We then rotate the sphere by 5° and quantify the net force needed to keep the sphere indented at a constant 150 µm. The results show that adhesion between sphere and the substrate does not noticeably affect the indentation force. It increases by only about 0.35% (Supplementary Fig. S7). This suggests that the effect of adhesion or friction between the sphere and the soft substrate has negligible effect on the measurement of elastic modulus by E-Pen.

### Application: measuring elastic modulus of soft tissue in vivo

**Figure 5:**
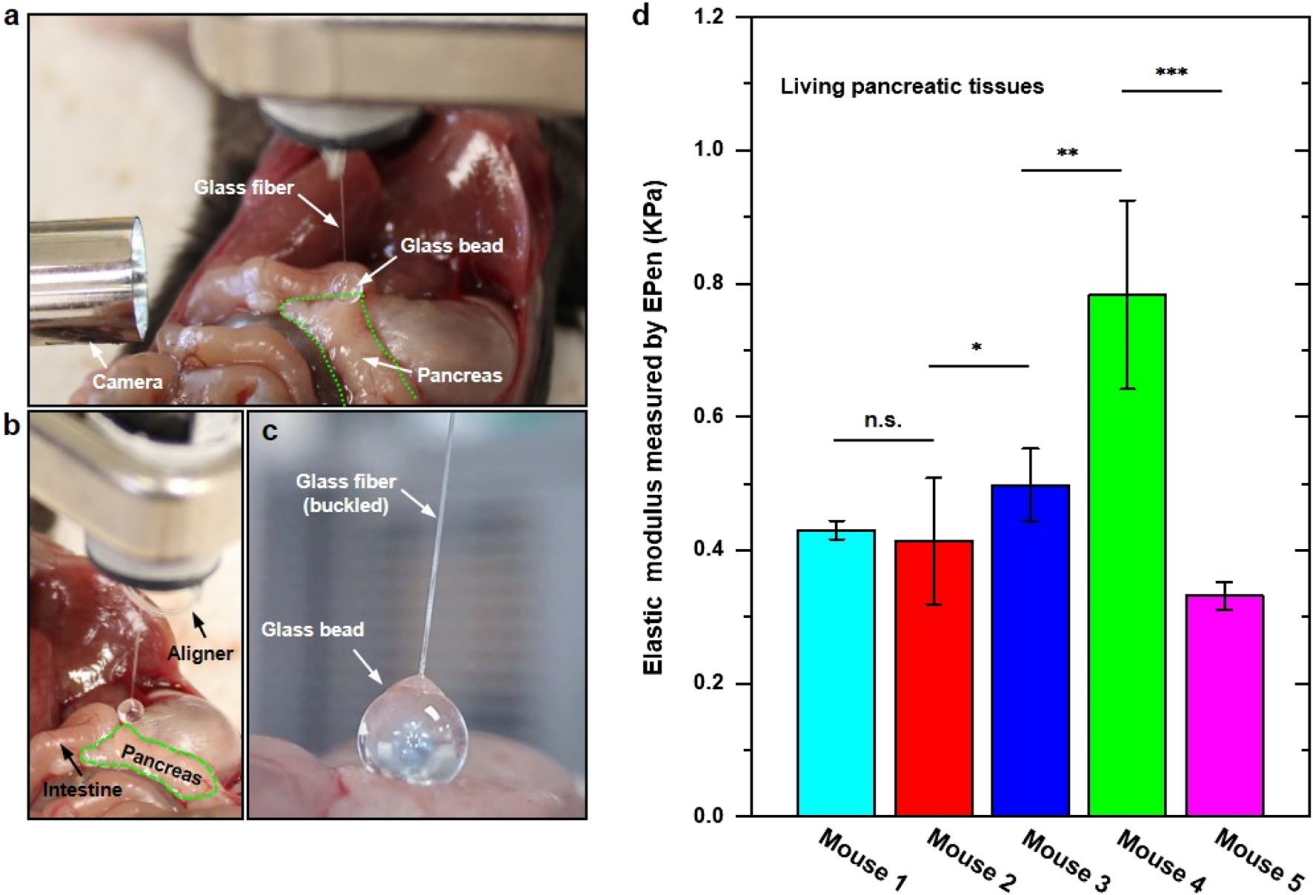
Application: measuring mouse pancreas stiffness. (**a**–**c**) Optical images of the EPen used for stiffness measurements of the soft pancreas tissue in vivo. (**d**) Measured elastic modulus of healthy and cancerous mouse pancreas by EPen. Data represent mean ± standard deviation. n = 3 EPen measurements for mouse 1 and 5, n = 11 EPen measurements for mouse 2, n = 9 EPen measurements for mouse 3, and n = 7 EPen measurements for mouse 4. Statistical significance is assessed using a two-tailed unpaired t-test (*p < 0.05, **p < 0.005, ***p < 0.0005).

We demonstrate the application of EPen to measure the elastic modulus of pancreatic tissues of healthy and KPC mice in vivo, where the green dotted area indicates the pancreatic tissue (Fig. 5a,b). The detailed description for each mouse could be found in the Methods section. To perform testing, the EPen is held vertically by a clamp with a camera fixed on the side view to monitor the contact of glass sphere with the tissue. The Vernier micrometer is used to induce the buckling of glass fiber and indenting the tissue surface to extract the elastic modulus of the pancreatic tissue in vivo (Fig. 5c). The measured elastic modulus of healthy and cancerous mouse pancreas by EPen are given in Fig. 5d. Mouse 1 is a control and has a healthy looking pancreas with the measured stiffness of 0.43 kPa. Mouse 2 and 3 show no statistically significant difference with the measured stiffness of 0.41 and 0.50 kPa respectively, which match with the fact that they are in early stages of disease. Interestingly, we notice that the mouse 4, which is reported “clearly diseased” by the clinic specialist, shows a perceptible increase in the stiffness of 0.78 kPa. Mouse 5 exhibits the smallest stiffness of 0.33 kPa, which match the fact that it has the youngest age (2 month old) and still in the early stage of disease without symptoms. Quantitative assessments of elastic modulus of mouse pancreas, healthy and cancerous, all of which surgically exposed but stayed intact in the live body confirm the potential clinical utility of the EPen.

## Discussion

A unique feature of our EPen, in contrast to existing linear spring-based method of measuring material behavior, is that the stiffness of EPen’s nonlinear spring (i.e., the buckling bar) is tunable. The underlying mechanics of nonlinear buckling and indentation embedded in the nature of the EPen device makes it capable for a wide range of in-situ stiffness measurement of soft materials or tissues. To bring this notion into a quantitative perspective, Eq. (4) gives

5 $$E_{s} \propto \frac{{d^{4} }}{{\sqrt r \cdot l^{2} \cdot \delta_{i}^{{{3 \mathord{\left/ {\vphantom {3 2}} \right. \kern-\nulldelimiterspace} 2}}} }}$$

where the glass fiber length $l$ and indentation amount $\delta_{i}$ can be considered as operational variables, sphere radius $r$ and fiber cross-sectional diameter $d$ as structural variables. All of these parameters influence the EPen’s measurement range of elastic modulus for soft materials or tissues. In the current design, the effective length $l$ for the buckling of the glass fiber can be easily tuned from 20 to 110 mm by means of an external magnet based on the needs. Glass fiber diameter $d$ and the glass bead radius $r$ can be chosen from 80 to 200 um, and from 1 to 3 mm respectively. And the indentation amount $\delta_{i}$ can vary within 0.05 to 0.3 mm in the actual tests. Therefore, the same EPen can measure elastic modulus of materials that span 3 orders of magnitude. For example, an 80 μm diameter glass fiber can be used to test materials with elastic modulus varying over three orders of magnitude (from 0.1 to 100 kPa). Larger glass fibers are used to measure the stiffer soft samples (Supplementary Fig. S8). Soft materials such as polydimethylsiloxane (PDMS)^36,37^ with elastic modulus as high as 4.2 MPa can also be tested by the same device by replacing the glass fiber of 200 μm diameter.

Measurement time for a modulus measurement is less than 1 min. It involves taking two measurements of the Vernier scale and two images by the buckling bar, before and after application of force on the sample. The measurement time can be significantly reduced to few seconds if the Vernier micrometer readings are done electronically. In that case no manual measurements will be necessary. Furthermore, the EPen is inexpensive, and it does not require any specialized facility for its assembly. It only requires a straight bar with known geometry and material properties so that the critical buckling load can be estimated, a Vernier micrometer to measure its end motion as it applies indentation on a sample. Owing to these beneficial characteristics, our EPen is suitable for measuring elastic modulus of soft tissues, particularly diseased ones for prognosis. The tissues can be in situ, intact with the body. However, the tissue needs to be accessible to the probe of the EPen. Hence, EPen is appropriate for use when the tissue is exposed during surgery or after it is resected (e.g., human colon tumor^38^).

To summarize, we have developed a hand-held pen type instrument (EPen: Elastic modulus Pen) to measure the local elastic modulus of soft materials and tissues on site, where the tip of the pen serves as the modulus probe. To investigate the accuracy and reliability of the EPen measurements, we provide a systematic experimental, computational and analytical studies of the method and demonstrate that it can be used as an accurate reliable tool for rapidly measuring the mechanical properties of soft materials and tissues. Assessments of the elastic modulus of mouse pancreas, healthy and cancerous, surgically exposed but attached to the body further confirm the clinical utility of the EPen. Our findings establish routes for the design and fabrication of stiffness measurement instruments that meet requirements for clinical use.

## Methods

### Assembly of glass bead-fiber

In the EPen, the glass bead is glued to the glass fiber. A glass rod with 80 µm diameter is firstly obtained by stripping off plastic coating from a fiber optic cable (Supplementary Fig. S1a). And a 10 cm long segment of the glass fiber is then cut out transversely using fiber cleaver (Supplementary Fig. S1b). A 3D printed aluminum funnel fixture with a hollow glass needle is used to align the fiber and the bead (Supplementary Fig. S1c). A glass sphere with 2 mm in diameter is placed on the funnel hole and glued to the glass fiber at the cut end, such that the fixture holds the sphere in a conical cavity. Finally, the glass sphere and glass fiber are assembled and glued together, guided by a 3D-printed fixture (Supplementary Fig. S1c). The fabricated product of glass bead-glass fiber assembly is shown in Supplementary Fig. S1e.

### Fabrication of the EPen

The end of glass bead-glass fiber assembly is glued to a spring-based piston enclosed into an aluminum tube that is secured to the body of the EPen (Supplementary Fig. S2). The forward motion of the piston is driven and controlled by a Vernier Micrometer (SM-13LH) attached to the EPen body, while the back spring (Supplementary Fig. S2c) allows the piston to return to initial state after the micrometer motion is reversed. Two concentric glass tube and metal tube are used to ensure the alignment and translation of glass fiber along the axial direction, while the inner tube also anchored to a stainless steel piston through its central hole. The translation of this piston (Supplementary Fig. S2d,e) is controlled by an external magnet that can freely move from outside the EPen glass enclosure, which thus can move the metal tube inside the concentric glass tube, and hence creating an adjustable length of the glass fiber for the buckling. A pair of arc-shaped wires are attached to the end side of EPen body to guide the direction and plane of buckling of the glass fiber.

### The operation of EPen

In the measurements, the pen is held vertically by using a clamp. The samples are placed on a movable z-stage which brings the sample close to the glass bead. The Vernier micrometer is used to bring the glass bead in contact with the sample, and then move the glass fiber farther until the fiber buckles. Two measurements of the Vernier micrometer by the buckling bar before and after application of force on the sample give the total translation $\delta_{{\text{m}}}$ of the glass fiber. A camera aligned orthogonally to the plane of arc-shaped aligner is used to visualize the fine motion of the glass bead and monitor the contact. The buckling angle $\theta$ is obtained based on two optical images of glass fiber in proximity of samples surface taken by the camera before and after the buckling.

### PA gel preparation

Polyacrylamide (PA) gels are synthesized following a protocol by Tse and Engler^35^. Acrylamide (40% sol., Sigma-Aldrich), N, N′- Methylenebis-acrylamide (2% sol., Sigma-Aldrich) and Dulbecco’s Phosphate Buffered Saline (DPBS, Corning) are mixed at different ratios according to the protocol for various Young’s moduli (0.5, 2.5 and 5 kPa). 1% (v/v) of ammonium persulfate (APS) solution (10% w/v APS, Bio-Rad) and 0.1% (v/v) of Tetramethylethylenediamine (TEMED) solution (Bio-Rad) is used to catalyze the polymerization reaction in the gel precursor solution. The gel precursor is then poured into a mold and a large coverless (Fisher Scientific) is placed on top to form a plane test surface. After polymerization for about 20 min, the coverglass is removed and the gel was rinsed 3 times with Dulbecco’s phosphate-buffered saline (DPBS).

### Finite element analysis

Three-dimensional finite element model (FEM) is used to perform the buckling and postbuckling analysis of long slender glass fiber and investigate how the buckling force changes at any post buckled state (Fig. 3e). Buckling analysis is firstly performed to determine the critical buckling loads and corresponding buckling modes of the long slender glass fiber with one end is pinned and the other end fully fixed. The first buckling mode is then used as initial small geometrical imperfection to trigger the buckling. The beam elements (B21 in Abaqus) are used to discretize the geometry of glass fiber with the length of 50 mm and diameter 80 μm. A total of 21,000 elements are used to ensure mesh convergence. FEA results quantitively give the buckling forces at any post buckled states with different buckling angles (Supplementary Fig. S4).

A 2D axisymmetric finite element model is further established to simulate the spherical indentation on PA gel substrate and study the indentation mechanics. For simulation, four-node bilinear axisymmetric quadrilateral (CAX4) elements are used to discretize the geometry of PA gel substrate with the thickness of 3 and 5 mm. Refined meshes are adopted to ensure the accuracy. The Young’s modulus and Poisson’s ratio used for PA gel are $E$ = 0.5, 2.5 or 5 kPa and ν = 0.49. The glass bead with the diameter of 2 mm is considered to be rigid. “Hard contact” is implemented to simulate the interface between the glass bead and the PA gel substrate. Displacement boundary conditions are assigned to the rigid glass bead to apply different levels of compression. In addition, the effect of interaction property, adhesive or frictionless, between the glass sphere and PA gel substrate is also studied. FEA results show that friction does not affect the indentation force (Supplementary Fig. S6).

### Description of mice

All animal procedures were approved by Institutional Animal Care and Use Committee (IACUC) at the University of Illinois and were conducted following the Guide for the Care and Use of Laboratory Animals. *Mouse 1*: 9 month old female HK2^fl/+^ (no modification of allele in the absence of Cre; wild type control) with a body weight of 30.4 g. Having previously collected these control mice, their pancreas is histologically indistinct from *wild type* mice. *Mouse 2*: 10 month old p48-Cre/LSL-Kras^G12D^/Akt2^fl/+^ female with a body weight of 20.6 g. p48Cre and LSL-Kras^G12D^ positive (KC mice^39^) and heterozygous for Akt2 as determined by PCR genotyping. This mouse has a mild morphological phenotype in regards to its pancreas where mutant KRAS alters tissue architecture, though there could be some protective effect from reduced AKT2 to suppress disease development. *Mouse 3*: 9 month old p48-Cre/LSL-Kras/Akt2^fl/+^ male with a body weight of 31.5 g. It has the same genotype as the mouse 2, with mild/slight pancreas stiffness. *Mouse 4*: 5.5 month old p48-Cre/LSL-Kras^G12D^/LSL-p53^R172H/+^ (KPC^40^) female with a body weight of 25.5 g. This mouse is PCR positive for both p48Cre and LSL-Kras and heterozygous for one mutant LSL-p53^R172H^ allele. KPC mice are the gold standard of mouse models of pancreatic cancer with a distinct cancer phenotype in the pancreas that resembles human pancreatic cancer on both morphological and molecular levels^40^. *Mouse 5*: 2 month old KPC male (no body weight available) that is Pdx1-Cre and LSL-Kras^G12D^ positive and heterozygous for LSL-p53^G12D^. This mouse has a more mild phenotype but will likely have worse diseased pancreas within 2–3 months. These mice were selected to include the control mouse 1, which has a floxed allele that is not recombined. Ideally, this would have been Akt2, but those mice were not generated, and HK2 served as its substitute. The next two mice (2 and 3) are KC mice with loss of one allele of Akt2, which does have a histotype/phenotype similar to KC mice, and we remain interested in how loss of this allele may impact tissue stiffness. The final two mice (4 and 5) are KPC mice at older and younger ages to determine the relative stiffness during the progression of disease.

## Supplementary Information

Supplementary Information

## Data Availability

The authors declare that all data supporting the findings of this study are available in the paper and its Supplementary Information.
