# Supplementary material for: A portable pen-sized instrumentation to measure stiffness of soft tissues in vivo
Source: Sci Rep. 2021 Jan 11;11:378. doi: 10.1038/s41598-020-79735-8 (PMC7801673; doi:10.1038/s41598-020-79735-8)
Supplement: Supplementary file 1 — Supplementary Information [file 41598_2020_79735_MOESM1_ESM.docx]

Supplementary Information for

**A portable pen-sized instrumentation to measure stiffness of soft tissues in vivo**

Zhengwei Li^1,4^, Alireza Tofangchi^1,4^, Robert Stavins^1^, Emon Bashar^1^, Ronald D. McKinney^2^, Paul J. Grippo^2^ and M. Taher A. Saif^1,3*^

*^1^Department of Mechanical Science and Engineering, University of Illinois at Urbana-Champaign, Urbana, IL, USA.*

*^2^Department of Medicine, University of Illinois at Chicago, Chicago, IL, USA.*

*^3^Department of Bioengineering, University of Illinois at Urbana-Champaign, Urbana, IL, USA.*

^4^These authors contributed equally to this work

^*^Correspondence and requests for materials should be addressed to M.T.A.S ([saif@illinois.edu](mailto:saif@illinois.edu))

**Table of Contents**

**Supplementary Notes………………………………………………………………..…………...**1

Supplementary note 1: Analytical modeling for buckling and postbuckling analysis………...……1

Supplementary note 2: Determination of parameter$\delta_{\text{b}}$ …….…………………………….……….4

Supplementary note 3: Effect of axial shortening of buckling bar…………………………………5

Supplementary note 4: Effect of sample thickness on the stiffness measurements ……………….6

**Supplementary Figures…………………………………………………………………………..**7

Supplementary Fig. S1. The assembly of the glass fiber and glass bead…..……………….……....7

Supplementary Fig. S2. Schematic design of the EPen…………………………………...………..8

Supplementary Fig. S3. Analytical model for the buckling and postbuckling analysis.…...…….....9

Supplementary Fig. S4. FEA for the buckling and postbuckling analysis. …...................……….10

Supplementary Fig. S5. FEA for the spherical indentation …...………….………………………11

Supplementary Fig. S6. FEA for investigating the friction effect during the indentation.……..….12

Supplementary Fig. S7. EPen measurements for the flexible polymer………………....……..….13

**Supplementary References...…………………………………………...………………………**14

**Supplementary Notes**

**Supplementary Note 1: Analytical model for the buckling and postbuckling analysis**

The buckling of a long glass fiber is utilized to quantify the applied force on the soft materials or tissues during the stiffness measurements. When the glass fiber continues to buckle with a rotation angle $\theta$, the applied force $P$ exceeds the critical buckling force with a small increase of force . Therefore, it will introduce a small error in the measurements when the critical buckling force is considered as the applied force $P$ at any post-buckling state. To quantitatively study how the applied force $P$changes with the buckling angle$\theta$, an analytical model is established for the buckling analysis of long slender glass fiber.

Consider a long bar of length $l$ with one end fully fixed and the other end held by a movable piston, as shown in Supplementary Fig. 3. Here, it should be made clear that the top end corresponds to the end of the glass fiber fixed to the glass sphere in the EPen. The relationship between bending moment $M$ and rotation angle $\theta$ can be expressed as,

$$\frac{M}{EI}=\frac{1}{\rho}=\frac{d\theta}{ds}=\frac{y"}{\sqrt{1-y'^{2}}}$$

where$\frac{d\theta}{ds}<0$. When $\left|\frac{d\theta}{ds}\right|\ll1$, then$\frac{d\theta}{ds}=\frac{y"}{\sqrt{1-y'^{2}}}\approx y"$ . Force balance gives$M=Py-Rx$. Therefore, the above equations can be further expressed as

$$y"+k^{2}y=b^{2}x$$

Where,$k^{2}=P/EI$and$b^{2}=R/EI$. The solution to this equation is obtained as

$$y=A\sin(kx)+B\cos(kx)+\frac{b^{2}}{k^{2}}x$$

Boundary conditions are $y(0)=0$and$y(l)=y'(l)=0$, which gives $B=0$ and

$$A\sin(kl)+\frac{b^{2}}{k^{2}}l=0$$

$$Ak\cos(kl)+\frac{b^{2}}{k^{2}}=0$$

From the equation (3) and (4), we get

$$\tan(kl)=kl$$

$$\frac{b^{2}}{k^{2}}=\frac{R}{P}=\alpha\frac{A}{l}$$

Here, $kl=4.493$and$\alpha=-kl\cos(kl)=0.9778$. Therefore, the equation (3) can be further expressed as,

$$y=A\alpha(\frac{x}{l}+\frac{1}{\alpha}\sin(kx))$$

Where, only the parameter $A$ is unknown.

In fact, the postbuckling deformation of the glass fiber is determined by the nonlinear moment-curvature equation (1). In order to find the relationship between $\theta$ and A, we expand the right side of equation (1) and retain the first two terms,

$$-\frac{Py}{EI}+\frac{Rx}{EI}=y"(1+\frac{y'^{2}}{2})$$

Assume there is no change on the glass fiber length under the applied force $P$. Therefore, the above equation can be further expressed as

$$-\frac{Py}{EI}+\frac{P}{EI}\alpha\frac{A}{l}\int_{0}^{s} (1-\frac{y'^{2}}{2})ds=y"(1+\frac{y'^{2}}{2})$$

Where we used $x=\int_{0}^{s} (1-\frac{y'^{2}}{2})ds$ and from equation (7): $\frac{R}{P}=\alpha\frac{A}{l}$. Let the approximate solution for equation (10) be

$$y_{a}=A\alpha\left[\frac{s}{l}+\frac{1}{\alpha}\sin(ks)\right]$$

Use of *y_a_(s)* in Eq. 10 gives a residual error:

If$y_{a}=y_{exact}$ , then$R(s)=0$. Orthogonalize $R(s)$ with the form^1^$g(s)=\frac{s}{l}+\frac{1}{\alpha}\sin(ks)$, that is

$$\int_{0}^{L} R(s)g(s)=0$$

Based on the above equation, the relationship between applied force $P$ and the critical buckling force is given by

$$\frac{P}{P_{cr}}=\frac{b_{1}\eta^{2}+b_{2}}{b_{3}\eta^{2}+b_{4}}\cdot\frac{1}{\gamma}$$

Where,$b_{1}=25.0376$, $b_{2}=9.61633$, $b_{3}=0.379736$, $b_{4}=0.476363$,$\gamma=20.142$ and $\eta=\frac{A}{l}$.

The buckling angle $\theta$ can be expressed as

$$\sin\theta=\left.\frac{dy}{ds}\right|_{s=0}=A\left(\frac{\alpha}{l}+k\right)$$

Substitute $\alpha=-kl\cos(kl)$into the equation (15), the buckling angle can be further given by

$$\sin\theta=Ak\left[1-\cos\left(kl\right)\right]$$

Thus, $\eta=\frac{A}{l}=\frac{\sin\theta}{kl\left[1-\cos\left(kl\right)\right]}\text{=}0.18\cdot\sin\theta$.

**Supplementary Note 2: Determination of parameter** $\delta_{\text{b}}$

In the EPen measurments, the total displacement $\delta_{\text{m}}$ of glass fiber could be directly read out by taking two measurements of the Vernier micrometer when the glass sphere initially contacts the sample and when the glass fiber buckles. Although it is impractical to measure the indentation amount $\delta_{i}$ on the soft materials or tissues, yet $\delta_{i}$ can be obtained based on the equation, where $\delta_{b}$ is the part of the translation that merely causes the buckling (Fig. 1b) and can be determined from the deformed shape of the glass fiber after buckling (Supplementary Fig. 3). The projection of buckled glass fiber along the x direction is

$$x=\int_{0}^{l} \sqrt{1-y'^{2}}ds$$

Assuming the conservation of length of the bar before and after buckling, then $\delta_{b}$ is given by

$$\delta_{b}=l-\int_{0}^{l} \sqrt{1-y'^{2}}ds$$

Use the shape equation$y=A\alpha(\frac{s}{l}+\frac{1}{\alpha}\sin(ks))$, $\delta_{b}$ can be rewritten as

$$\delta_{b}=\frac{1}{2}A^{2}\alpha^{2}\left[\frac{1}{l}+\frac{2\sin\left(kl\right)}{l\alpha}+\frac{k^{2}l}{2\alpha^{2}}+\frac{k}{4\alpha^{2}}\cdot\sin\left(2kl\right)\right]$$

Using Eq. (16) in (19), we obtain the relation between $\delta_{b}$ and $\theta$

$$\frac{\delta_{b}}{\sin^{2}\theta}=\frac{\alpha^{2}l}{2(kl)^{2}\left(1-\cos\left(kl\right)\right)^{2}}\left[1+\frac{2\sin\left(kl\right)}{\alpha}+\frac{(kl)^{2}}{2\alpha^{2}}+\frac{(kl)}{4\alpha^{2}}\cdot\sin\left(2kl\right)\right]$$

Substituting the constant parameters of $kl=4.493$and $\alpha=0.9778$ into the above equation results in the final expression as

$$\delta_{b}=0.16\cdot l\sin^{2}\theta$$

**Supplementary Note 3: Effect of axial shortening of buckling bar**

We ignored the effect of axial elastic shortening of the buckling bar while calculating $\delta_{b}$ (Fig. 1a-c). This is justified, since elastic shortening ($\delta_{e}$~$l(r/l)^{2}$) is about two orders of magnitude less than$\delta_{b}$~$l(D/l)^{2}$, where $r$ is the radius of the buckling bar (i.e., 40 µm), $D$is a measure of transverse buckling after indentation in the soft material. Thus, $\delta_{e}/\delta_{b}$~$\left(r/D\right)^{2}$. In our case, $r$=40 µm, and $D$> 1 mm, giving $\delta_{e}/\delta_{b}$~$10^{-3}$, i.e., $\delta_{e}$ is about 3 orders of magnitude smaller than $\delta_{b}$. Hence ignoring $\delta_{e}$ compared to $\delta_{b}$ is reasonable.

**Supplementary Note 4: Effect of sample thickness on the stiffness measurements**

The thickness of the gel sample is chosen such that the effect of the well boundaries on the elastic modulus is minimized. Since the 3D printed well is made from hard material and the PA gel is adhered to the wall and the bottom of the well, the indentation induced by glass bead of the EPen is reduced compared to that of a free large volume of a similar gel sample. Thus, the gel sample appears stiffer. Such effect can be minimized by choosing smaller sphere and/or minimizing the depth of indentation. We choose glass sphere radius $R$=1 mm as a compromise. Smaller sphere often penetrates soft materials (e.g., 2 kPa gel), while the buckling length of the glass fiber needs to be larger (e.g. $l$>10 cm) to lower the buckling load. In order to verify the effect of indentation depth on the measured modulus, we carry out a finite element analysis of indentation on a soft substrate bounded by a rigid boundary, which is similar to that of the experimental well. The measured modulus becomes independent of the rigid boundaries as long as the well diameter,$D/2>5R$, and the indentation depth is within 5% of the depth of the well (Supplementary Fig. 4).

**Supplementary Figures**


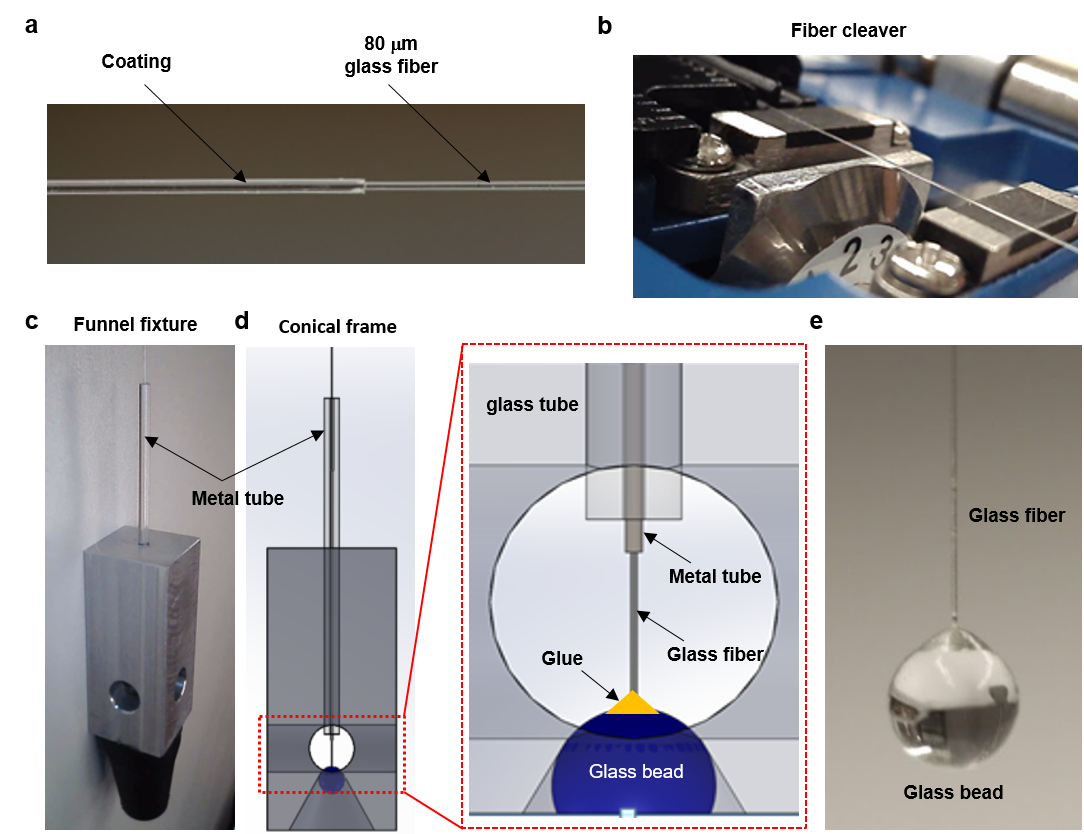


**Supplementary Fig. 1**. **The assembly of the glass fiber and glass bead. a**, Optical image of fiber optic cable consisting of a glass fiber with 80 μm in diameter and a plastic coating layer. **b**, Optical image of the fiber cleaver used to cut the glass fiber transversely. **c**, 3D printed aluminum fixture used for glass fiber-bead assembly, where the glass fiber aligned by a 32-GA tube and glued to the glass bead at one end. **d,** Schematics of the cross-section of conical frame (drawn by using AutoCAD 2017, https://www.autodesk.com). **e**, Optical image of a glass fiber with an 80 μm diameter glued to a glass bead with 2 mm spherical diameter.


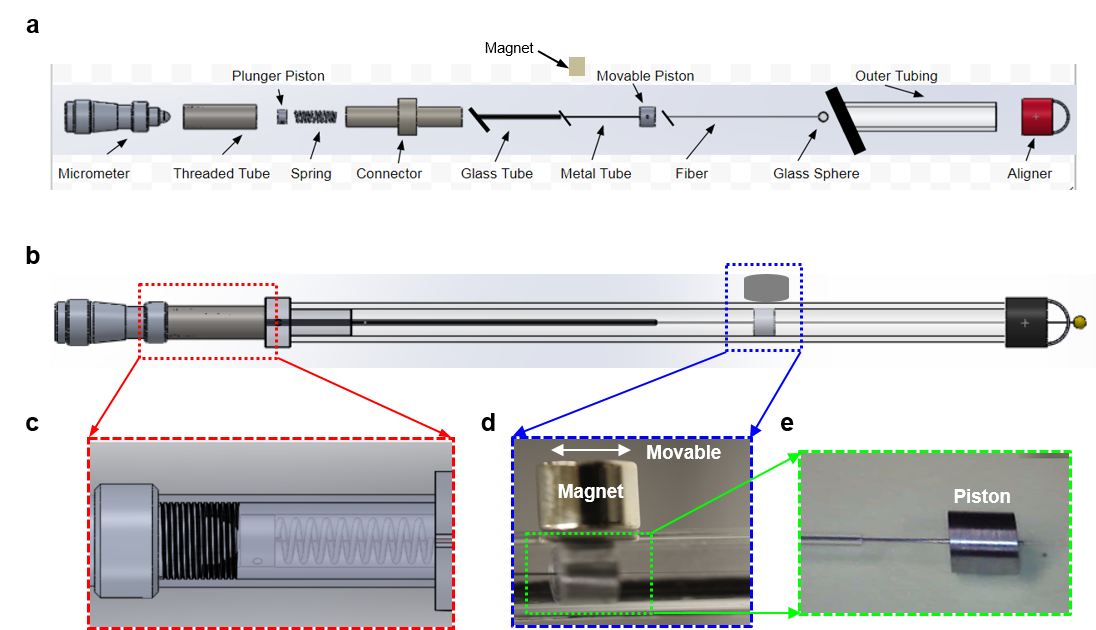


**Supplementary Fig. 2**. **Schematic design of the EPen. a**, Schematics of the EPen with each component indicated. **b**, Schematic of the fabricated EPen. **c**, Schematic of the spring loaded piston, which position can be precisely controlled by a vernier micrometer (**a-c** were drawn by using AutoCAD 2017, <https://www.autodesk.com>). **d, e**, The optical images of moveable piston controlled by an external magnet to adjust the effective length of glass fiber.


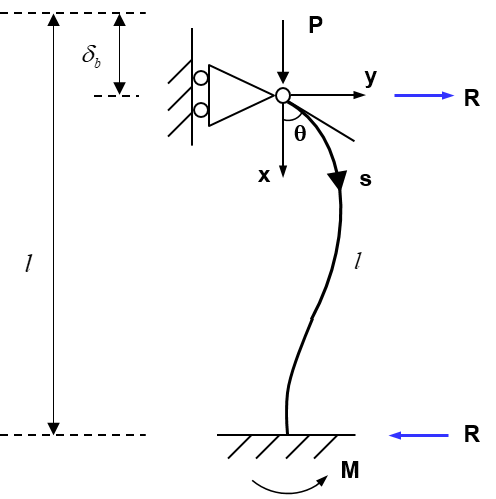


**Supplementary Fig. 3**. **Analytical model for the buckling and postbuckling analysis.**


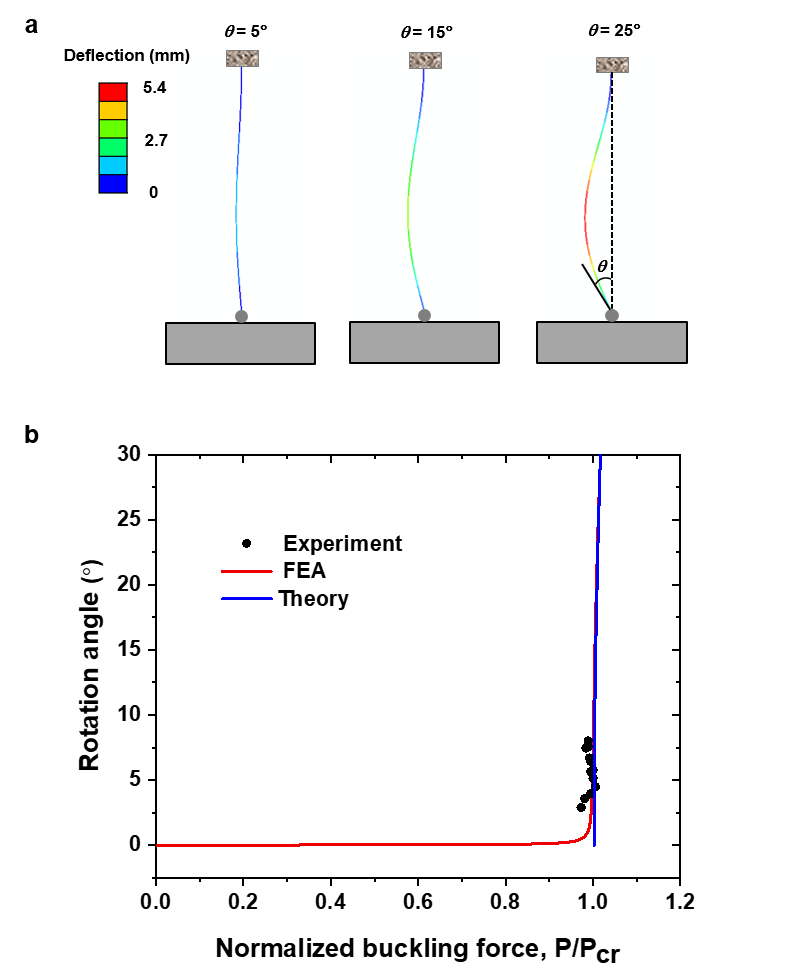


**Supplementary Fig. 4**. **FEA for the buckling and postbuckling analysis. a**, Deflection of buckled glass fiber with various rotation buckling angles, where one end of the glass fiber is fully fixed and the other end pinned. **b**, Measurements of applied force as a function of buckling rotation angles obtained from the experiment (black dots) are quantitatively consistent with analytical calculations of buckling mechanics (analytical; blue line) and finite element analysis (FEA, red line).


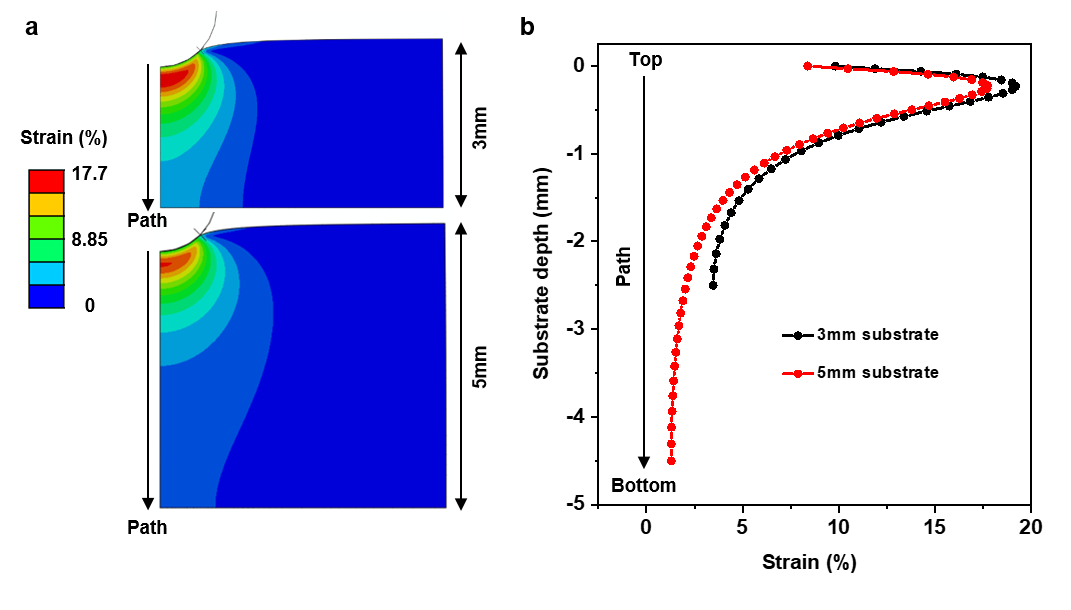
**Supplementary Fig. 5**. **FEA for the spherical indentation. a**, Strain contours of PA gel substrate with 3 mm and 5 mm thickness respectively under the spherical indentation. The young’s modulus of substrate is 5 kPa and the indentation depth is 500 μm. **b**, Strain distributions along the depth (as indicated in **a**) of PA gel substrate with 3 mm and 5 mm thickness respectively.

**
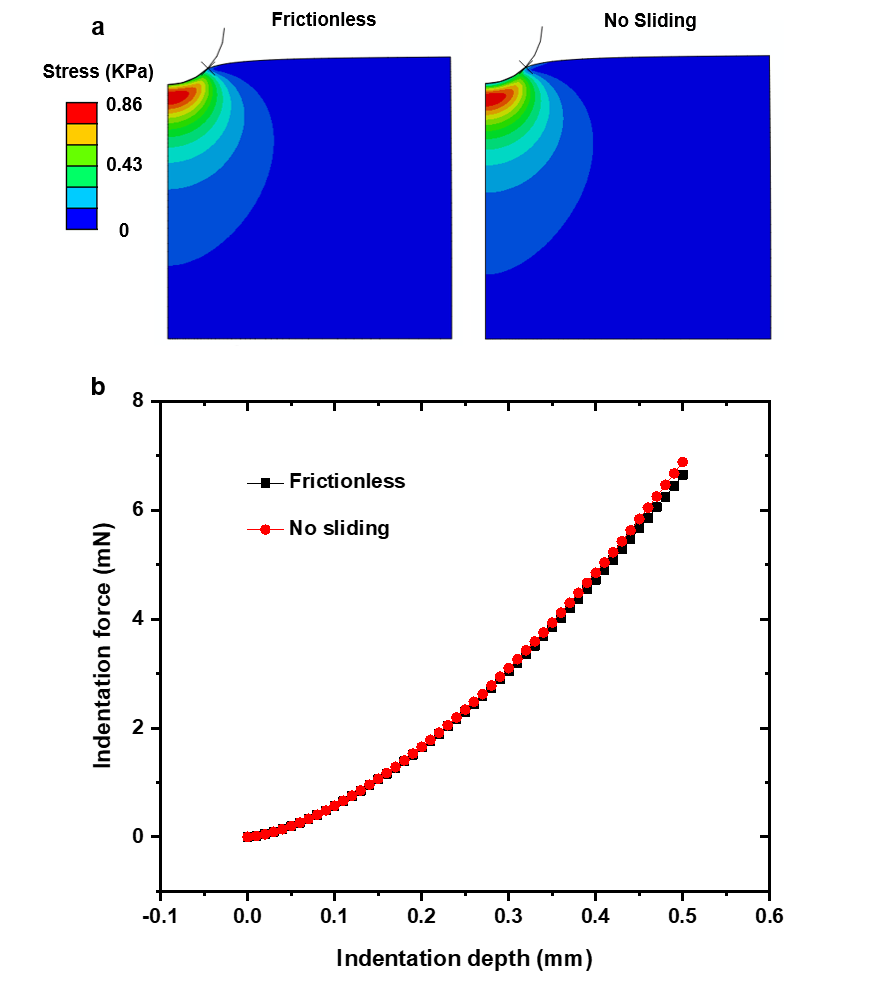
**

**Supplementary Fig. 6**. **FEA to investigate the effect of friction or adhesion on the indentation load. a**, Stress contours of PA gel substrate under 500 µm spherical indentation with the contact properties of “frictionless” and “no sliding” between the glass bead and PA gel substrate respectively. **b**, Net indentation force versus spherical indentation depth for “frictionless” and “no sliding” contacts respectively. Here, “frictionless” contact allows the sliding between the glass bead and PA gel substrate, but “no sliding” contact does not allow the sliding or separation.


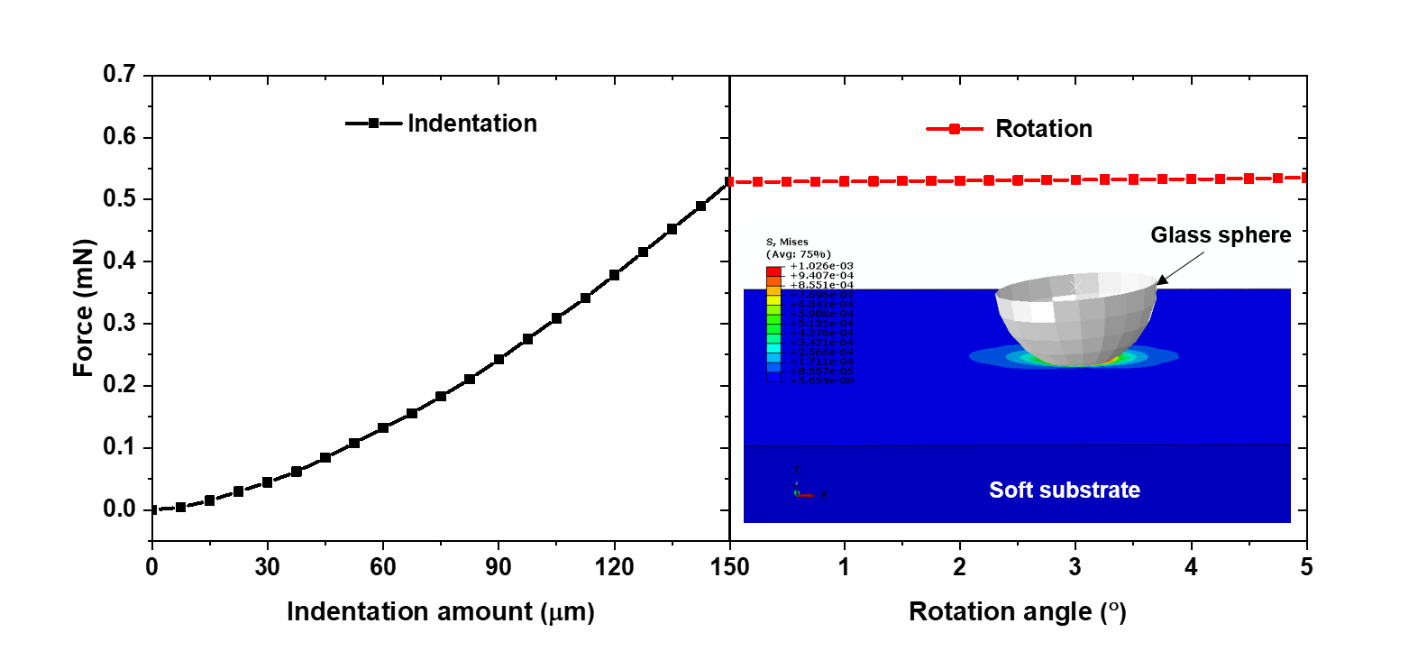


**Supplementary Fig. 7**. **3D** **FEA to investigate the effect of interfacial adhesion between the glass sphere and the substrate on the indentation force.** 2 mm diameter glass sphere is used to indent 5 KPa soft substrate and then rotated by 5° under a no-slip contact condition. The indentation force increases negligibly during the rotation. The insert shows the stress contours of soft substrate after 5° rotation of glass sphere.


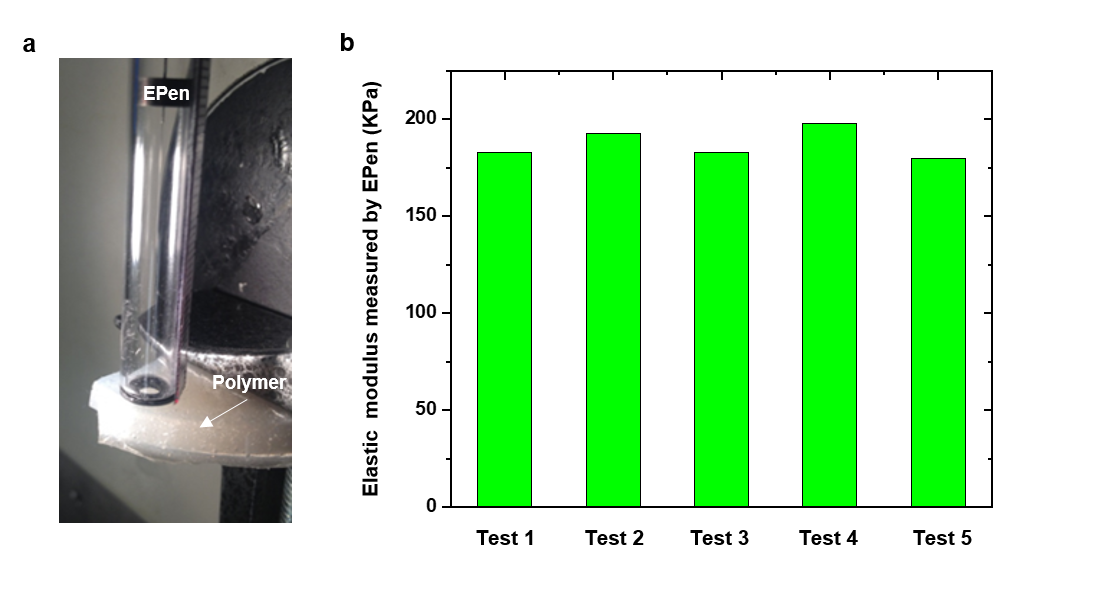


**Supplementary Fig. 8**. **EPen** **measurements for the flexible polymer. a**, Optical image of the EPen measurements on the flexible polymer by using 120 μm diameter glass fiber. **b**, Measured elastic modulus of the flexible polymer by EPen at the different tests. The measured average elastic modulus is 188 kPa which is close to the commercial value 200 kPa given in manual.

**Supplementary References**

1. Saif, M.T.A. On a tunable bistable MEMS-theory and experiment. *J. Microelectromech. Syst.* **9**,157-170 (2000).
